# The Relationship Between the Fibrinogen-to-Albumin Ratio and Short-Term Mortality in Chinese Patients With Chronic Heart Failure: A Retrospective Cohort Analysis

**DOI:** 10.1155/crp/9292002

**Published:** 2025-09-22

**Authors:** Lei Lei Guo, Ping Liu, Li Na Cai, Li Hu, Yue Shan Zhou

**Affiliations:** ^1^Department of Cardiology, Jiangyou People's Hospital, Jiangyou 621700, Sichuan, China; ^2^Department of Anesthesiology, Jiangyou People's Hospital, Jiangyou 621700, Sichuan, China

**Keywords:** albumin, fibrinogen, fibrinogen-to-albumin ratio, mortality

## Abstract

**Background:** Recent studies have identified an association between the fibrinogen-to-albumin ratio (FAR) and the prognosis of coronary heart disease; however, evidence regarding its significance in heart failure patients remains limited. This study aims to examine the relationship between the FAR and short-term mortality among individuals with heart failure.

**Methods:** In this retrospective cohort study, we conducted an analysis of clinical data from patients with heart failure who were hospitalized at Zigong Fourth People's Hospital from December 2016 to June 2019. The primary exposure variable was the FAR, while the outcomes of interest were mortality rates at 28 days and 3 months. Multivariate logistic regression evaluated FAR's independent association with short-term mortality, with predictive accuracy assessed via ROC curves and subgroup consistency through stratified analyses. Furthermore, smooth curve fitting was utilized to investigate the linear relationship, and a series of sensitivity analyses were conducted to validate the robustness of the findings.

**Results:** The analysis included 1880 participants, of whom 58.1% were females and 54.1% were aged 60–80 years. Our study showed that a one standard deviation rise in the FAR was linked to a 45% increase in 28-day mortality (OR = 1.45, 95% CI = 1.02–2.06, *p*=0.04) after adjusting for potential confounding factors. The 28-day mortality rate was markedly elevated in the high FAR group (FAR > 0.126) compared to the low FAR group (OR = 4.01, 95% CI = 1.17–13.82, *p*=0.028). Comparable findings were noted at the 3-month mark. There were no significant interactions found in the subgroup analysis. A linear association was identified between FAR and short-term mortality. The optimal FAR cutoff value for predicting 28-day mortality was 0.156 (sensitivity 68.0%, specificity 59.4%, AUC = 0.654), while for 3-month mortality, it was 0.156 (sensitivity 68.0%, specificity 58.3%, AUC = 0.647). Sensitivity analyses corroborated the robustness of our findings.

**Conclusion:** A positive correlation exists between the FAR and short-term mortality among Chinese patients with heart failure. These findings underscore the necessity for further investigation into the underlying pathophysiological mechanisms and potential therapeutic interventions associated with FAR in the context of heart failure.

## 1. Introduction

Congestive heart failure (CHF) represents a clinical syndrome indicative of the advanced stages of various cardiovascular diseases (CVDs). It is characterized by high prevalence and mortality rates, resulting in decreased functional capacity and quality of life [1]. With an estimated impact on over 64 million individuals worldwide, the global burden of heart failure continues to increase [2]. The management of patients with heart failure presents a considerable challenge, requiring the proactive identification of emerging risk factors and the formulation of effective treatment strategies.

Fibrinogen, a glycoprotein complex produced in the liver, plays a role in inflammation and thrombosis [3–5]. This biomarker is correlated with an elevated risk of CVDs and has been associated with increased mortality risk in individuals with coronary artery disease [6, 7]. Xu et al. [8] demonstrated that elevated blood fibrinogen levels are associated with an increased likelihood of readmission within 6 months in individuals with heart failure, while not affecting mortality risk.

Albumin levels, as a marker of nutritional status [9], have been linked to negative clinical outcomes in various CVDs [10]. Hypoalbuminemia frequently occurs in individuals with heart failure and may arise due to chronic inflammation, infection, and malnutrition [11]. Kato et al. [12] demonstrated that elevated serum albumin levels serve as a protective factor against both in-hospital and long-term mortality in patients with heart failure.

Systemic inflammation plays a crucial role in the development of heart failure and acts as a predictor of negative outcomes [13]. The fibrinogen-to-albumin ratio (FAR) is a newly combined inflammation parameter that has been shown to increase mortality and worsen prognosis in patients with cancer, stroke, and sepsis [14–16]. Furthermore, it has been correlated with all-cause mortality among patients experiencing acute myocardial infarction and heart failure [17, 18]. A recent study indicated that the FAR might serve as a more effective prognostic indicator than albumin or fibrinogen alone in cases of heart failure. Nonetheless, there exists a notable deficiency in research exploring the association between FAR levels and short-term mortality among patients with heart failure. The objective of this retrospective study was to evaluate the association between the FAR and short-term mortality among Chinese patients diagnosed with heart failure.

## 2. Methods

### 2.1. Data Source

This study utilized the central database of Zigong Fourth People's Hospital in Sichuan, China, in conjunction with data from PhysioNet, to analyze the characteristics of 2008 adults diagnosed with CHF between December 2016 and June 2019 [19]. The database was created through the consolidation of electronic health records and subsequent monitoring of patient progress [20]. The study employed a retrospective cohort design and analyzed 166 data attributes to ensure data validity. The attributes encompassed demographic data, comorbid conditions, laboratory findings, clinical outcomes, and therapeutic interventions for patients diagnosed with heart failure. To safeguard privacy, all data were anonymized as of June 8, 2020. Upon successful completion of the mandatory NIH web-based training course and certification (Certification No. 12631985), the author was granted access to the database. Ethical approval for the study was secured from the Ethics Committee of Zigong Fourth People's Hospital (Approval No. 2020-010) [20]. Due to the retrospective nature of the study, informed consent was waived. The research was conducted in accordance with the Strengthening the Reporting of Observational Studies in Epidemiology (STROBE) guidelines.

### 2.2. Study Population

**Figure 1:**
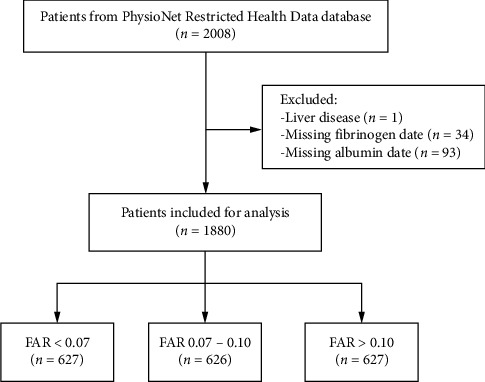
Flowchart of patient selection.

We selected all patients diagnosed with heart failure from this database who had information on mortality within 28 days and 3 months. A total of 845 men and 1163 women were included in the database in 2008. The study followed the Helsinki Declaration. Patients were diagnosed with heart failure based on the criteria established by the European Society of Cardiology (ESC) [21]. The exclusion criteria for this study were as follows: (1) live disease, (2) missing fibrinogen data, and (3) missing albumin data (Figure 1).

### 2.3. Data Collection

The data retrieved from the database encompass variables including age, sex, respiratory rate, heart rate, systolic blood pressure (SBP), diastolic blood pressure (DBP), and body mass index (BMI), in addition to mortality information within 28 days and 3 months. The study considered comorbidities such as the New York Heart Association (NYHA) cardiac function classification, type of heart failure, diabetes, and chronic kidney disease. Laboratory assessments included measurements of white blood cell (WBC) count, red blood cell (RBC) count, platelet count, activated partial thromboplastin time (APTT), thrombin time (TT), international normalized ratio (INR), fibrinogen levels, creatinine, uric acid, estimated glomerular filtration rate (eGFR), albumin, alanine aminotransferase (ALT), aspartate transaminase (AST), indirect bilirubin, direct bilirubin, cholesterol, triglycerides, and high-density lipoprotein cholesterol (HDL-C). Demographic data were collected during the patient's initial hospital admission, while all other data were gathered on the first day of hospitalization. The prescribed medications included aspirin, statins, renin–angiotensin–aldosterone system inhibitors/angiotensin receptor blockers (RAAS/ARBs), diuretics, and spironolactone.

### 2.4. Covariates

We evaluated potential confounding covariates by taking into account prior research findings, clinical relevance, and the frequency of outcome events [22, 23]. We included the following variables: age, gender, heart rate, SBP, DBP, BMI, WBC, creatinine, BNP, sodium, uric acid, LVEF, AST, diabetes, chronic kidney disease, diuretics, spironolactone, and RAAS/ARBs.

### 2.5. Power Analysis

We used the statistical program G∗Power to determine the required sample size and conduct the statistical power analysis for this study. Based on a population sample size of 1879, the analysis yielded a statistical power exceeding 99% at a significance level of *p* < 0.05, indicating sufficient power for this sample size.

### 2.6. FAR Assessment

Fibrinogen and albumin concentrations are expressed in grams per liter (g/L). The FAR is determined by dividing the concentration of fibrinogen by that of albumin.

### 2.7. Primary Outcome

The primary endpoint was cardiovascular mortality at 28 days and 3 months.

### 2.8. Statistical Analysis

For continuous variables, the mean ± standard deviation was reported for data exhibiting normal distributions, whereas the median was utilized for data with skewed distributions. Categorical variables were described using frequencies or percentages. Student's *t*-test was employed for normally distributed variables, while the Mann–Whitney *U* test was applied to variables with skewed distributions. Nominal data were analyzed using the chi-square test, and Bonferroni's post hoc analysis was conducted to evaluate differences among groups. The patients with heart failure were divided into three groups based on their FAR levels: *Q*_1_: FAR < 0.07, *Q*_2_: FAR 0.07–0.10, and *Q*_3_: FAR > 0.10. We standardized the FAR using Z-scores and then included it in multivariate logistic analyses to investigate the impact of a one standard deviation increase (per SD) in the FAR on cardiovascular mortality. Confounding factors presented a significant challenge in the multivariate analysis. To address this issue, a range of statistical models were utilized to enhance the robustness of the findings. Specifically, a multivariate-adjusted restricted cubic spline model with four knots was constructed to fit the odds ratio (OR) curves, facilitating the examination of a potential nonlinear dose–response relationship between FAR and mortality. We examined the relationship between three distinct models and the primary outcome events utilizing multivariate logistic regression analysis: (1) Model 1, which was adjusted for age and gender; (2) Model 2, which included additional adjustments for heart rate, SBP, DBP, BMI, WBC count, creatinine, B-type natriuretic peptide (BNP), sodium, uric acid, left ventricular ejection fraction (LVEF), and AST; and (3) Model 3, which further incorporated adjustments for diabetes, chronic kidney disease, use of diuretics, spironolactone, and RAAS/ARBs. The optimal cutoff values of FAR for predicting 28-day and 3-month mortality were determined using receiver-operating characteristic (ROC) curve analysis, with corresponding sensitivity and specificity calculated.

### 2.9. Subgroup Analysis and Sensitivity Analysis

The subgroups were examined utilizing a stratified binary logistic regression model. Subgroup analyses were performed on covariates including gender, NYHA cardiac function classification, diabetes, chronic kidney disease, diuretics, and spironolactone to further investigate the impact of these covariates (FAR per standard deviation) on outcome events. To evaluate heterogeneity across various subgroups, multivariate logistic regression was employed. Additionally, likelihood ratio tests were conducted to assess interactions among these subgroups.

A series of sensitivity analyses [24] was conducted to evaluate the robustness of our research findings. First, our data had the following missing values (Table S1). More than half of the patients in the cohort had no recorded LVEF values (missing percentage: 68.37%). Directly using LVEF as a covariate would have led to significant missing data. Therefore, we included the presence or absence of LVEF values as a covariate in our models (Table 1 and Table S2). We applied a rigorous statistical approach to manage missing data by implementing multiple imputation [25] with five iterations, employing the chained equation technique available in the R mice package. Subsequently, the imputed dataset was analyzed using multivariate logistic regression to validate the robustness of the findings.

Second, by calculating E-values [26], we assessed the potential for unmeasured confounding between FAR and mortality within 28 days and 3 months.

The statistical analyses were conducted using R Version 3.3.2 (https://www.Rproject.org, The R Foundation) and Free Statistics software Version 1.9.2. Statistical significance was set at a two-sided *p* value of < 0.05.

## 3. Results

### 3.1. Baseline Characteristics of Patients With CHF

The study cohort comprised 1880 patients diagnosed with heart failure. Among these individuals, 54.1% were aged between 60 and 80 years, and 58.1% were females. The mortality rates observed were 1.7% at 28 days and 1.9% at 3 months. The baseline characteristics of the cohorts, stratified according to the FAR, are presented in Table 1. The median FAR was 0.1, with median fibrinogen and albumin levels of 3.2 ± 1.0 and 36.6 ± 5.0 g/L, respectively. Patients with higher FAR demonstrated increased levels of heart rate, respiratory rate, WBC, platelet count, APTT, creatinine, triglycerides, BNP, high-sensitivity troponin, and hs-CRP, along with higher frequencies of aldosterone and diuretic use (*p* < 0.05). In contrast, individuals in the lower FAR group exhibited increased levels of RBC, TT, AST, ALT, indirect bilirubin, direct bilirubin, HDL-C, eGFR, chloride, and sodium (*p* < 0.05). No significant differences were noted among groups in terms of age, gender, BMI, type of heart failure, or NYHA classification (*p* > 0.05).

### 3.2. Association Between FAR and Clinical Outcomes

**Figure 2:**
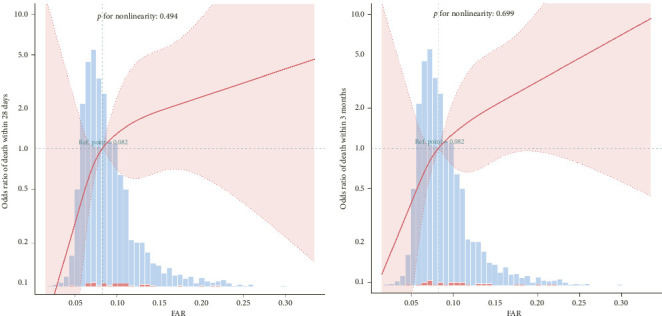
Linear relationship between fibrinogen-to-albumin ratio (FAR) and mortality in 28 days (a), in 3 months (b). Adjustment factors included age, gender, heart rate, systolic blood pressure; diastolic blood pressure, body mass index, white blood cell, creatinine, brain natriuretic peptide, sodium, uric acid, left ventricular ejection fraction (LVEF), aspartate transaminase, diabetes, chronic kidney disease, diuretics, spironolactone, angiotensin-converting enzyme inhibitor/angiotensin receptor blocker. The red line and pink area represent the estimated values and their corresponding 95% confidence intervals, respectively.

A linear relationship was observed between the FAR and short-term mortality within 28 days, at 3 months in heart failure patients after adjusting for some covariates (Figure 2).

**Figure 3:**
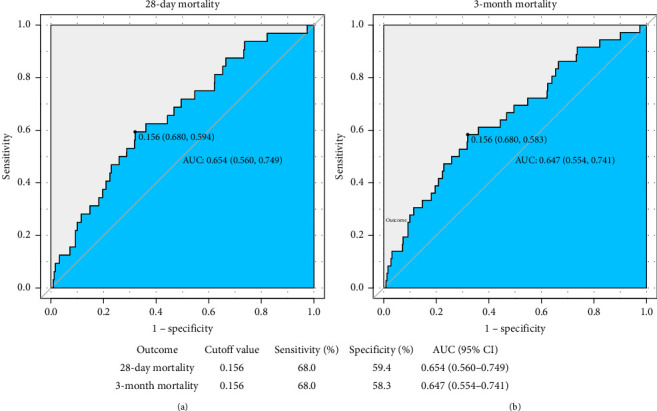
ROC curve analysis for 28-day (Figure (a)) and 30-day (Figure (b)) in-hospital mortality: The blue curve represents the ROC curve of the fibrinogen-to-albumin ratio (FAR), along with its sensitivity/specificity, AUC (95% CI), and cutoff values for predicting 28-day and 30-day mortality.

In multivariable logistic regression analyses, FAR (per SD) were positively associated with short-term mortality in all models when the FAR (per SD) was analyzed as a continuous variable (in 28 days OR = 1.45, 95% confidence interval [CI] = 1.02–2.06, *p*=0.04; in 3 months OR = 1.51,95% CI = 1.09–2.08, *p*=0.012). In Model 3, when the FAR was analyzed as tertiles, the highest tertile (Q3) compared to the lowest tertile (Q1) was significantly associated with an increased risk of mortality after controlling for potential confounding variables. The adjusted OR for mortality at 28 days was 4.01 (95% CI = 1.17–13.82, *p*=0.028), and at 3 months, the adjusted OR was 3.59 (95% CI = 1.19–10.83, *p*=0.023) (refer to Table 2). ROC analysis demonstrated modest predictive accuracy of FAR for both 28-day mortality (AUC 0.654, 95% CI 0.560–0.749) and 3-month mortality (AUC 0.647, 95% CI 0.554–0.741). At the optimal cutoff value of 0.156, sensitivity reached 68.0% for both time points, while specificity was 59.4% for 28-day and 58.3% for 3-month mortality (Figure 3).

### 3.3. Subgroup Analysis by Adjusted Potential Effect Confounders

**Figure 4:**
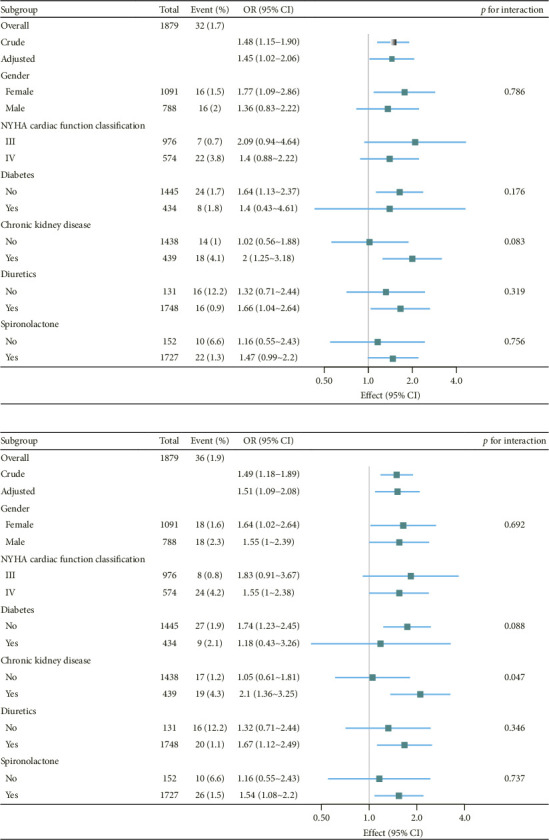
Subgroup analysis of relationships between fibrinogen-to-albumin ratio (per SD) and short-term mortality in hospitalized patients with congestive heart failure. Odds ratios (ORs) were adjusted for age, heart rate, systolic blood pressure; diastolic blood pressure, body mass index, white blood cell, creatinine, brain natriuretic peptide, sodium, uric acid, left ventricular ejection fraction (LVEF), aspartate transaminase, angiotensin-converting enzyme inhibitor/angiotensin receptor blocker. Note: (a) the relationship between the fibrinogen-to-albumin ratio (FAR) and 28-day mortality and (b) the relationship between FAR and 3-month mortality. These data represent the results after removing the maximum outlier from the FAR dataset (1879 cases).

Figure 4 presents the stratified analysis of the associations between the FAR per standard deviation and short-term mortality. The subgroup analysis indicated no significant evidence of effect modification or interaction among common risk factors for mortality within 28 days and 3 months, as all *p* values for interaction exceeded 0.05.

### 3.4. Sensitivity Analyses

To enhance the robustness of our findings, we conducted a series of sensitivity analyses. Initially, a sensitivity analysis employing a multivariable logistic regression model was undertaken in patients, following the imputation of missing covariate values. The association between the FAR (per standard deviation) and short-term mortality was consistently observed, with a 28-day OR of 1.26 (95% CI: 0.89–1.80, *p*=0.192) and a 3-month OR of 1.38 (95% CI: 1.02–1.89, *p*=0.039), as presented in Table S2. These results indicate that the overall trend remained stable. Furthermore, we computed an E-value to evaluate the sensitivity to unmeasured confounding. For 28-day and 3-month mortality outcomes, the E-values corresponding to unmeasured confounding were 2.26 and 2.39, respectively.

## 4. Discussion

To our knowledge, this retrospective cohort study is the first to conclusively establish that the FAR is independently correlated with increased short-term mortality in patients with heart failure. The application of smooth curve fitting techniques revealed a linear relationship, and similar association patterns were observed across subgroup analyses. At the cutoff value of 0.156, FAR demonstrated moderately high sensitivity and specificity in predicting short-term mortality, suggesting its utility as a practical indicator for risk stratification in heart failure. Various sensitivity analyses further corroborated the robustness of these findings. These results may have significant implications for the management of heart failure, particularly within East Asian populations.

Serum albumin is an extensively researched biomarker in hospitalized patients, playing a vital role in the acute inflammatory response [27]. Prior research has established hypoproteinemia as an independent risk factor for mortality among individuals with heart failure [11]. Furthermore, albumin acts as a crucial indicator for evaluating nutritional status. The interaction between nutritional status and chronic inflammation contributes to decreased albumin levels in patients with heart failure [9]. Decreases in serum albumin levels are associated with disease advancement. In contrast, increased albumin levels during hospitalization have been associated with a decreased risk of all-cause mortality and hospitalization within 1 year in patients with heart failure [12].

Fibrinogen is integral to numerous pathological processes [28]. Numerous clinical studies have demonstrated a correlation between elevated blood fibrinogen levels and an increased risk of CVDs [29]. Schulze et al. [30] demonstrated that elevated blood fibrinogen levels are correlated with a 15% increased risk of CVD. Furthermore, a separate meta-analysis revealed that an increase of 1 g/L in fibrinogen concentration is associated with a 142% heightened risk of coronary artery disease [31]. Collectively, these studies suggest a significant association between elevated blood fibrinogen concentrations and both all-cause mortality and cardiac mortality. Xu et al. [8] discovered a U-shaped correlation between fibrinogen levels and the probability of hospital readmission in patients with heart failure. Specifically, the study found that when fibrinogen levels reached 2.4 g/L, heart failure patients had the highest risk of rehospitalization. Interestingly, the study did not find any significant association between fibrinogen levels and the risk of mortality within 6 months.

Originally, FAR was introduced as a new inflammatory marker that has been shown to increase mortality rates and worsen prognosis in patients with cancer, stroke, COVID-19, and sepsis [5, 14, 15, 32]. Li et al. [33] identified the FAR as a significant risk factor for the development of coronary artery disease, recognizing it as an emerging biomarker for inflammation and thrombosis. Furthermore, FAR has been validated as an independent prognostic indicator for major adverse cardiovascular events (MACEs) at 30 days, 6 months, and 1 year following the implantation of drug-eluting stents (DES), with hazard ratios of 1.095 (95% CI: 1.011–1.186; *p*=0.025), 1.076 (95% CI: 1.009–1.147; *p*=0.026), and 1.080 (95% CI: 1.022–1.141; *p*=0.006), respectively. In a prospective cohort study [34], the group with elevated FAR demonstrated increased mortality rates and adverse cardiovascular events in comparison with the group with lower FAR, suggesting that FAR serves as a significant predictor of negative outcomes in patients with coronary artery disease. Although the aforementioned studies predominantly focus on the association between FAR and mortality as well as adverse outcomes, there is a notable paucity of research investigating the relationship between FAR and mortality in the context of heart failure.

Sawatani et al. [18] demonstrated that an elevated FAR is independently correlated with diabetes and ischemic heart disease, contributing to increased 730-day mortality in patients with acute decompensated heart failure, in comparison with those with a low FAR. Similarly, Huang et al. [23] identified a significant association between high FAR and an elevated risk of major adverse cardiac and cerebral events (MACCEs), particularly among patients with diabetes. Collectively, these findings underscore the role of FAR as an independent risk factor for adverse clinical outcomes in individuals with acute decompensated heart failure, with a pronounced impact on those with concurrent diabetes.

Yang et al. [22] identified that an elevated FAR in patients with chronic heart failure significantly increases the risk of mortality and functions as an independent prognostic indicator, with an optimal threshold value of 9.06. A retrospective analysis utilizing the MIMIC-IV database corroborated these findings, demonstrating that FAR is a strong independent predictor of both 90-day and 1-year mortality, as well as the length of hospital stay in heart failure patients, outperforming the predictive capabilities of fibrinogen and albumin individually [35]. This study posits that the relationship between elevated FAR and adverse outcomes in heart failure may be attributed to underlying inflammatory processes and a prothrombotic state. It is crucial to acknowledge, however, that this particular study solely examined all-cause mortality and lacked data on cardiovascular mortality and heart failure hospitalizations.

Drawing upon prior research, our study reveals that an elevated FAR is significantly correlated with an increased risk of short-term cardiovascular mortality among Chinese adult patients with heart failure. This finding indicates that FAR may serve as a valuable prognostic marker for short-term cardiovascular mortality. Further sensitivity analyses corroborate the robustness of these results. Our research contributes critical insights into the management and control of heart failure, offering promising implications for future prevention strategies and therapeutic interventions.

The FAR cutoff in this study differs from previous reports [17, 18, 22, 23] mainly related to inconsistent unit definitions and population heterogeneity, and also associated with differences in disease severity and comorbidities. Therefore, clinical interpretation requires considering both unit standardization and population characteristics.

The exact mechanisms that connect higher FAR levels to increase short-term mortality have not been completely understood, and it is possible that inflammation and prothrombotic state are involved [3, 27, 36]. The association between FAR and short-term mortality in heart failure may be due to similar inflammatory disease mechanisms, leading to increased fibrinogen and decreased albumin. In summary, higher levels of inflammation and prothrombotic state are indicated by higher FAR, both of which increase short-term mortality of heart failure.

This study presents several notable advantages. First, the sample size is comparatively large relative to previous studies of a similar nature. Second, while prior research has predominantly concentrated on examining the association between the FAR and long-term all-cause mortality, as well as cardiovascular mortality in heart failure, our study uniquely endeavors to elucidate the relationship between FAR and short-term cardiovascular mortality in heart failure. Third, we perform a sensitivity analysis to evaluate the potential influence of unmeasured confounding variables.

Several limitations of this study warrant consideration. First, as a single-center retrospective investigation, the potential for selection bias may compromise the accuracy of our findings. Consequently, multicenter studies are necessary to validate these results. Second, the presence of missing data for certain variables could potentially distort the outcomes. However, we conducted a series of sensitivity analyses, which affirm the robustness of our findings. Third, this study exclusively utilized the FAR value from the initial measurement and did not account for its temporal dynamics. Fourth, due to the substantial missing data on LVEF, our study encountered difficulties in differentiating between reduced and preserved ejection fraction heart failure.

## 5. Conclusions

The FAR has been identified as a factor associated with an elevated risk of short-term mortality in patients with heart failure. To substantiate and enhance the robustness of these findings, further large-scale prospective studies are warranted.

## Figures and Tables

**Table 1:** Baseline characteristics of the patients with congestive heart failure.

Variables	Total (*n* = 1880)	FAR			*p* value
		T1 (< 0.07) (*n* = 627)	T2 (0.07–0.10) (*n* = 626)	T3 (> 0.1) (*n* = 627)	
Demographics					
Age, *n* (%)					0.819
< 60	167 (8.9)	60 (9.6)	49 (7.8)	58 (9.3)	
60–80	1017 (54.1)	341 (54.4)	341 (54.5)	335 (53.4)	
> 80	696 (37.0)	226 (36)	236 (37.7)	234 (37.3)	
Gender, *n* (%)					0.424
Female	1092 (58.1)	377 (60.1)	360 (57.5)	355 (56.6)	
Male	788 (41.9)	250 (39.9)	266 (42.5)	272 (43.4)	
Heart rate (beats/min)	85.2 ± 21.4	84.4 ± 22.2	84.2 ± 21.3	87.1 ± 20.7	0.03
Respiration (beats/min)	19.1 ± 1.7	19.0 ± 1.7	19.1 ± 1.7	19.2 ± 1.8	0.038
SBP (mmHg)	131.5 ± 24.7	130.1 ± 24.5	131.6 ± 24.2	132.8 ± 25.3	0.154
DBP (mmHg)	76.7 ± 14.4	77.7 ± 13.9	76.8 ± 15.1	75.6 ± 14.3	0.027
BMI, kg/m^2^	21.9 ± 14.1	21.5 ± 6.0	22.3 ± 22.0	21.8 ± 8.7	0.585
Comorbidities					
NYHA, *n* (%)					0.432
II	329 (17.5)	102 (16.3)	120 (19.2)	107 (17.1)	
III	976 (51.9)	337 (53.7)	324 (51.8)	315 (50.2)	
IV	575 (30.6)	188 (30)	182 (29.1)	205 (32.7)	
Type of heart failure, *n* (%)					0.747
Both	1392 (74.0)	473 (75.4)	463 (74)	456 (72.7)	
Left	438 (23.3)	137 (21.9)	149 (23.8)	152 (24.2)	
Right	50 (2.7)	17 (2.7)	14 (2.2)	19 (3)	
Diabetes, *n* (%)					< 0.001
No	1445 (76.9)	518 (82.6)	485 (77.5)	442 (70.5)	
Yes	435 (23.1)	109 (17.4)	141 (22.5)	185 (29.5)	
Chronic kidney disease, *n* (%)					< 0.001
No	1439 (76.6)	511 (81.5)	487 (77.9)	441 (70.4)	
Yes	439 (23.4)	116 (18.5)	138 (22.1)	185 (29.6)	
Laboratory parameters					
White blood cell (10^9^/L)	7.3 ± 3.4	6.4 ± 2.9	6.9 ± 3.0	8.5 ± 4.0	< 0.001
Red blood cell (10^12^/L)	3.9 ± 0.8	3.9 ± 0.7	3.9 ± 0.7	3.7 ± 0.8	< 0.001
Platelet (10^9^/L)	144.7 ± 64.4	129.7 ± 58.2	140.0 ± 54.2	164.1 ± 74.2	< 0.001
APTT (S)	35.4 ± 8.2	34.9 ± 6.7	34.9 ± 8.2	36.2 ± 9.5	0.006
TT (S)	17.2 ± 5.6	17.8 ± 3.0	17.2 ± 4.8	16.6 ± 7.9	< 0.001
INR (%)	1.3 ± 0.7	1.4 ± 0.5	1.4 ± 1.0	1.3 ± 0.6	0.332
Fibrinogen (g/L)	3.2 ± 1.0	2.3 ± 0.4	3.0 ± 0.4	4.3 ± 1.0	< 0.001
Albumin (g/L)	36.6 ± 5.0	38.6 ± 4.6	37.1 ± 4.2	34.0 ± 4.9	< 0.001
FAR (%)	0.1 ± 0.0	0.1 ± 0.0	0.1 ± 0.0	0.1 ± 0.0	< 0.001
AST (U/L)	39.0 (22.0, 74.0)	44.0 (25.0, 81.0)	40.0 (22.0, 73.0)	35.0 (21.0, 68.0)	0.001
ALT (U/L)	21.0 (13.0, 36.0)	23.0 (15.0, 41.5)	22.0 (14.0, 36.0)	18.0 (12.0, 31.0)	< 0.001
Indirect bilirubin (μmol/L)	11.7 (7.8, 17.1)	14.2 (9.9, 20.2)	11.6 (7.8, 17.1)	9.3 (6.4, 13.6)	< 0.001
Direct bilirubin (μmol/L)	6.5 (4.1, 10.5)	7.6 (5.0, 13.1)	6.6 (4.2, 10.6)	5.3 (3.4, 8.5)	< 0.001
Cholesterol (mmol/L)	3.6 (3.0, 4.3)	3.7 (2.9, 4.3)	3.6 (3.0, 4.3)	3.6 (3.0, 4.3)	0.922
Triglyceride (mmol/L)	1.0 (0.7, 1.3)	0.9 (0.7, 1.3)	0.9 (0.7, 1.2)	1.0 (0.7, 1.4)	0.009
LDL-C (mmol/L)	1.8 (1.3, 2.3)	1.8 (1.3, 2.3)	1.8 (1.4, 2.3)	1.7 (1.4, 2.3)	0.976
HDL-C (mmol/L)	1.1 (0.9, 1.3)	1.1 (0.9, 1.3)	1.1 (0.9, 1.3)	1.0 (0.8, 1.3)	0.012
Uric acid (mmol/L)	483.1 ± 167.5	482.5 ± 168.9	488.4 ± 167.1	478.5 ± 166.6	0.58
Creatinine (μmol/L)	87.2 (65.1, 122.8)	82.2 (63.0, 113.9)	86.1 (65.0, 115.0)	97.1 (68.2, 142.6)	< 0.001
eGFR (mL/min/1.73 m^2^)	64.6 (41.4, 89.7)	69.1 (48.3, 92.3)	66.6 (44.2, 88.6)	56.7 (34.5, 87.5)	< 0.001
BNP (pg/mL)	765.6 (315.2, 1758.3)	781.6 (328.6, 1751.3)	744.4 (336.9, 1745.1)	791.6 (291.0, 1761.0)	0.926
High-sensitivity troponin (pg/mL)	0.1 (0.0, 0.1)	0.0 (0.0, 0.1)	0.0 (0.0, 0.1)	0.1 (0.0, 0.1)	< 0.001
hs-CRP (mg/L)	9.4 (4.0, 29.3)	4.5 (2.4, 9.5)	8.2 (3.9, 18.7)	28.4 (10.0, 73.6)	< 0.001
Calcium (mmol/L)	2.3 ± 0.2	2.3 ± 0.2	2.3 ± 0.2	2.3 ± 0.2	0.012
Potassium (mmol/L)	4.0 ± 0.7	3.9 ± 0.6	4.0 ± 0.7	4.0 ± 0.8	0.087
Chloride (mmol/L)	101.9 ± 5.9	102.6 ± 5.7	102.4 ± 5.3	100.8 ± 6.5	< 0.001
Sodium (mmol/L)	138.3 ± 4.8	139.0 ± 4.6	138.8 ± 4.4	137.2 ± 5.2	< 0.001
LVEF (%)	50.8 ± 13.2	49.3 ± 14.0	50.5 ± 13.5	52.5 ± 11.8	0.049
Medication use					
Aspirin, *n* (%)	915 (48.7)	275 (43.9)	307 (49)	333 (53.1)	0.005
ACEI/ARB, *n* (%)	729 (38.8)	266 (42.4)	235 (37.5)	228 (36.4)	0.065
Diuretics, *n* (%)	1749 (93.0)	591 (94.3)	589 (94.1)	569 (90.7)	0.023
Spironolactone, *n* (%)	1728 (91.9)	594 (94.7)	573 (91.5)	561 (89.5)	0.003
Statins, *n* (%)	774 (41.2)	229 (36.5)	255 (40.7)	290 (46.3)	0.002
Outcomes (death)					
28 days, *n* (%)	32 (1.7)	5 (0.8)	8 (1.3)	19 (3)	0.006
3 months, *n* (%)	36 (1.9)	6 (1)	9 (1.4)	21 (3.3)	0.005

*Note:* AST: alanine aminotransferase; ALT: aspartate transaminase; FAR: fibrinogen-to-albumin ratio; hs-CRP: high-sensitivity C-reactive protein; NYHA: New York Heart Association cardiac function classification.

Abbreviations: ACEI/ARB, angiotensin-converting enzyme inhibitor/angiotensin receptor blocker; APTT, activated partial thromboplastin time; BMI, body mass index; BNP, brain natriuretic peptide; DBP, diastolic blood pressure; eGFR, estimated glomerular filtration rate; HDL-C, high-density lipoprotein cholesterol; INR, international normalized ratio; LDL-C, low-density lipoprotein cholesterol; LVEF, left ventricular ejection fraction; SBP, systolic blood pressure; TT, thrombin time.

**Table 2:** Multivariate logistic regression analyses of FAR and clinical outcomes in patients with congestive heart failure.

Outcomes	Crude mode		Model 1		Model 2		Model 3	
	OR (95% CI)	*p* value	OR (95% CI)	*p* value	OR (95% CI)	*p* value	OR (95% CI)	*p* value
*Outcome of congestive HF 28 days of death*								
FAR (per 1 SD)	1.48 (1.15–1.90)	0.002	1.49 (1.15–1.93)	0.002	1.49 (1.07–2.06)	0.018	1.45 (1.02–2.06)	0.04
*T*_1_ (< −0.516)	1 (ref)		1 (ref)		1 (ref)		1 (ref)	
*T*_2_ (−0.516 to 0.126)	1.61 (0.52–4.95)	0.406	1.58 (0.51–4.87)	0.423	1.85 (0.54–6.36)	0.330	1.80 (0.50–6.54)	0.372
*T*_3_ (> 0.126)	3.89 (1.44–10.49)	0.007	3.83 (1.42–10.33)	0.008	3.92 (1.22–12.57)	0.021	4.01 (1.17–13.82)	0.028
*p* for trend		0.003		0.004		0.014		0.019

*Outcome of congestive HF 3 months of death*								
FAR (per 1 SD)	1.49 (1.18–1.89)	0.001	1.51 (1.18–1.93)	0.001	1.54 (1.14–2.10)	0.005	1.51 (1.09–2.08)	0.012
*T*_1_ (< −0.516)	1 (ref)		1 (ref)		1 (ref)		1 (ref)	
*T*_2_ (−0.516 to 0.126)	1.51 (0.53–4.27)	0.437	1.48 (0.52–4.20)	0.457	1.70 (0.55–5.24)	0.357	1.64 (0.51–5.26)	0.405
*T*_3_ (> 0.126)	3.59 (1.44–8.96)	0.006	3.54 (1.42–8.83)	0.007	3.61 (1.26–10.38)	0.017	3.59 (1.19–10.83)	0.023
*p* for trend		0.003		0.003		0.011		0.016

*Note:* These data represent the results after removing the maximum outlier from the FAR dataset (1879 cases). The crude model was adjusted for none. Model 1 was adjusted for age and gender. Model 2 was adjusted for Model 1+ (heart rate, systolic blood pressure; diastolic blood pressure, body mass index, white blood cell, creatinine, brain natriuretic peptide, sodium, uric acid, left ventricular ejection fraction, aspartate transaminase). Model 3 was adjusted for Model 2+ (diabetes, chronic kidney disease, diuretics, spironolactone, angiotensin-converting enzyme inhibitor/angiotensin receptor blocker).

## Data Availability

The dataset used in this retrospective cohort study is sourced from the publicly accessible Chinese population database, PhysioNet, which can be found at the following link: https://physionet.org/content/heart-failure-zigong/1.3/. Lei Lei Guo has successfully completed the educational course offered by the National Institutes of Health and received access to the database with certification number 12631985.

## Supporting Information

Supporting Information 1 Table S1: Distribution of missing values. This table provides a detailed breakdown of missing data points across all variables included in the study. It highlights the proportion and distribution of missing values, which is essential for understanding the data quality and the need for imputation.

Supporting Information 2 Table S2: After multiple imputation multivariate logistic regression analyses of FAR and death in patients with congestive heart failure. This table presents the results of multivariate logistic regression analyses performed after multiple imputation. It examines the association between the fibrinogen-to-albumin ratio (FAR) and mortality risk in patients with congestive heart failure, adjusting for relevant covariates.
