# Supplementary material for: The Relationship Between the Fibrinogen-to-Albumin Ratio and Short-Term Mortality in Chinese Patients With Chronic Heart Failure: A Retrospective Cohort Analysis
Source: Cardiol Res Pract. 2025 Sep 22;2025:9292002. doi: 10.1155/crp/9292002 (PMC12479150; doi:10.1155/crp/9292002)
Supplement: Supporting Information 1 — Table S1: Distribution of missing values. This table provides a detailed breakdown of missing data points across all variables included in the study. It highlights the proportion and distribution of missing values, which is essential for understanding the data quality and the need for imputation. [file 9292002.f1.docx]

**Table S1:** Distribution of missing values.

| Variable | Miss frequency | Miss percentage% |
| --- | --- | --- |
| Fibrinogen, (g/L) | 34 | 1.6932 |
| Albumin, (g/L) | 102 | 5.0797 |
| White blood cell, (10^9^/L) | 27 | 1.3446 |
| Red blood cell, (10^12^/L) | 27 | 1.3446 |
| platelet, (10^9^/L) | 27 | 1.3446 |
| Calcium, (mmol/L) | 11 | 0.5478 |
| Chloride, (mmol/L) | 11 | 0.5478 |
| Sodium, (mmol/L) | 11 | 0.5478 |
| Potassium, (mmol/L) | 11 | 0.5478 |
| AST, (U/L) | 102 | 5.0797 |
| ALT, (U/L) | 102 | 5.0797 |
| Direct bilirubin, (umol/L) | 102 | 5.0797 |
| Indirect bilirubin, (umol/L) | 102 | 5.0797 |
| Uric acid, (μmol/L) | 23 | 1.1454 |
| Creatinine, (μmol/L) | 23 | 1.1454 |
| eGFR (mL/min/1.73 m^2^) | 63 | 3.1375 |
| APTT, s | 34 | 1.6932 |
| Thrombin time, s | 34 | 1.6932 |
| International normalized ratio | 35 | 1.743 |
| Cholesterol, (mmol/l) | 198 | 9.8606 |
| LDL-C, (mmol/l) | 198 | 9.8606 |
| HDL-C, (mmol/l) | 198 | 9.8606 |
| Triglyceride, (mmol/l) | 198 | 9.8606 |
| Brain natriuretic peptide, (pg /mL) | 35 | 1.743 |
| Hs-CRP, (mg/L) | 1067 | 53.1375 |
| High sensitivity troponin, (pg /mL) | 79 | 3.9343 |
| LVEF, % | 1373 | 68.3765 |

**Abbreviations:** APTT activated partial thromboplastin time; BNP brain natriuretic peptide; HDL-C High-density lipoprotein cholesterol; LDL-C Low-density lipoprotein-C; AST Alanine aminotransferase; ALT Aspartate transaminase, hs-CRP high sensitivity protein; eGFR estimated glomerular filtration rate; LVEF Left Ventricular Ejection Fraction.
