# Supplementary material for: The Relationship Between the Fibrinogen-to-Albumin Ratio and Short-Term Mortality in Chinese Patients With Chronic Heart Failure: A Retrospective Cohort Analysis
Source: Cardiol Res Pract. 2025 Sep 22;2025:9292002. doi: 10.1155/crp/9292002 (PMC12479150; doi:10.1155/crp/9292002)
Supplement: Supporting Information 2 — Table S2: After multiple imputation multivariate logistic regression analyses of FAR and death in patients with congestive heart failure. This table presents the results of multivariate logistic regression analyses performed after multiple imputation. It examines the association between the fibrinogen-to-albumin ratio (FAR) and mortality risk in patients with congestive heart failure, adjusting for relevant covariates. [file 9292002.f2.docx]

**Table S2:** After multiple imputation multivariate logistic regression analyses of FAR and death in Patients with congestive Heart Failure.

| Outcomes | Crude mode |  | Model1 | | Model2 | | Model3 | |
| --- | --- | --- | --- | --- | --- | --- | --- | --- |
|  | OR (95%CI) | P value | OR (95%CI) | P value | OR (95%CI) | P value | OR (95%CI) | P value |
| **outcome of congestive HF 28 days death** | | | | | | | | |
| FAR (per 1 SD) | 1.37 (1.10-1.71) | 0.005 | 1.39 (1.10-1.74) | 0.005 | 1.37 (1.01-1.85) | 0.046 | 1.26 (0.89-1.80) | 0.192 |
| T_1_ (< -0.517) | 1 (Ref) |  | 1(Ref) |  | 1 (Ref) |  | 1 (Ref) |  |
| T_2_ ( -0.517 to 0.127) | 1.51 (0.53-4.26) | 0.439 | 1.48 (0.52-4.19) | 0.459 | 1.86 (0.59-5.83) | 0.286 | 1.77 (0.54-5.83) | 0.346 |
| T_3_ (>0.127) | 3.75 (1.51-9.31) | 0.004 | 3.69 (1.48-9.17) | 0.005 | 3.38 (1.16-9.83) | 0.026 | 2.97 (0.96-9.23) | 0.060 |
| P for trend |  | 0.002 |  | 0.002 |  | 0.019 |  | 0.052 |
| **outcome of congestive HF 3 months death** | | | | | |  |  |  |
| FAR (per 1 SD) | 1.37 (1.11-1.69) | 0.003 | 1.39 (1.12-1.74) | 0.003 | 1.45 (1.09-1.91) | 0.010 | 1.38 (1.02-1.89) | 0.039 |
| T_1_ (< -0.517) | 1 (Ref) |  | 1 (Ref) |  | 1 (Ref) |  | 1 (Ref) |  |
| T_2_ ( -0.517 to 0.127) | 1.13 (0.43-2.94) | 0.807 | 1.11 (0.43-2.90) | 0.831 | 1.24 (0.43-3.53) | 0.691 | 1.21 (0.37-8.23) | 0.733 |
| T_3_ (>0.127) | 3.20 (1.43-7.15) | 0.005 | 3.14 (1.41-7.03) | 0.005 | 2.99 (1.17-7.63) | 0.022 | 2.68 (1.00-7.17) | 0.049 |
| P for trend |  | 0.002 |  | 0.002 |  | 0.012 |  | 0.033 |

Crude model was adjusted for none

Model 1 was adjusted for age, gender.

Model 2 was adjusted for model 1+ (heart rate, systolic blood pressure; diastolic blood pressure, body mass index, White blood cell, Creatinine, brain natriuretic peptide, Sodium, uric acid, left ventricular ejection fraction, Aspartate transaminase).

Model 3 was adjusted for model 2+ (Diabetes, Chronic kidney disease, Diuretics, Spironolactone, angiotensin-converting enzyme inhibitor/angiotensin receptor blocker)
